# B-Comet Assay (Comet Assay on Buccal Cells) for the Evaluation of Primary DNA Damage in Human Biomonitoring Studies

**DOI:** 10.3390/ijerph17249234

**Published:** 2020-12-10

**Authors:** Carla Russo, Mattia Acito, Cristina Fatigoni, Milena Villarini, Massimo Moretti

**Affiliations:** Department of Pharmaceutical Sciences (Unit of Public Health), University of Perugia, Via del Giochetto, 06122 Perugia, Italy; carla.russo@studenti.unipg.it (C.R.); mattia.acito@studenti.unipg.it (M.A.); cristina.fatigoni@unipg.it (C.F.); massimo.moretti@unipg.it (M.M.)

**Keywords:** comet assay, DNA damage, buccal lymphocytes, human biomonitoring

## Abstract

Many subjects perceive venous blood collection as too invasive, and thus moving to better-accepted procedures for leukocytes collection might be crucial in human biomonitoring studies (e.g., biomonitoring of occupational or residential exposure to genotoxins) management. In this context, primary DNA damage was assessed in buccal lymphocytes (BLs), fresh whole venous, and capillary blood leukocytes, and compared with that in peripheral blood lymphocytes (PBLs)—the most frequently used cells—in 15 young subjects. Mouthwashes were collected after the volunteers rinsed their mouths with normal saline, and BLs were isolated by density gradient centrifugation. Blood samples were collected by venipuncture or by lancet. Anthropometric and lifestyle information was obtained by the administration of a structured questionnaire. As shown in the Bland-Altman plots, the level of agreement between BLs and PBLs lied within the accepted range, we thus enrolled a wider population (*n* = 54) to assess baseline DNA damage in BLs. In these cells, mean values of tail length (µm), tail intensity (%), and tail moment were 25.7 ± 0.9, 6.7 ± 0.4 and 1.0 ± 0.1, respectively. No significant association was observed between sex and smoking habit with any of the DNA damage parameters. Conversely, underweight subjects displayed significantly higher genomic instability compared with normal weight group (*p* < 0.05). In conclusion, we successfully managed to set up and update a non-invasive and well-accepted procedure for the isolation of BLs from saliva that could be useful in upcoming biomonitoring studies.

## 1. Introduction

According to the World Health Organization (WHO), human biomonitoring (HBM) can be defined as “the assessment of human exposure to chemicals or their effects by measuring these chemicals, their metabolites or reaction products in human specimens” [1]. HBM is a crucial tool as it provides, through the use of biomarkers—a portmanteau of biological markers—useful information on whether and/or to what extent environmental, occupational, and dietary exposure affects human population, thereby helping to identify risk distribution and health-policy making. Several definitions of biomarkers are reported in the literature; however, fortunately, they largely overlap. The WHO has proposed the following definition of biomarkers: “almost any measurement reflecting an interaction between a biological system and a potential hazard, which may be chemical, physical, or biological. The measured response may be functional and physiological, biochemical at the cellular level, or a molecular interaction” [2]. Measurement of biomarkers can be performed in a wide range of specimens, including blood, urine, saliva, faeces, sweat, hair, and nails [1]. Among others, DNA damage is widely recognized as an important biomarker, by virtue of its potential role in cell transformation and tumorigenesis [3].

Comet assay is a sensitive, rapid, flexible, and low-cost method for the measurement of primary DNA damage and repair in eukaryotic—and some prokaryotic—cells or disaggregated tissues, based on electrophoretic separation of DNA from nucleoids originating from single cells embedded in layers of agarose. Thanks to its versatility, it has been applied over time to several non-animal and animal species (both invertebrates and vertebrates), including humans [4,5,6,7]. In the context of HBM, comet assay is widely employed as either a biomarker of exposure (biologically effective dose) or effect (early biological effect), or in tandem with other early-effect biomarkers such as chromosome aberrations and micronuclei [8].

The standard alkaline version of the assay allows the measurement of DNA single- and double-strand breaks, as well as alkali-labile sites (expressed as frank-strand breaks in the alkaline test conditions) and transient breaks induced by DNA repair enzymes [9]. Moreover, the enzyme-modified version of the assay is also able to detect a number of DNA base modifications, which are converted into breaks by incubation with lesion-specific endonucleases [10,11,12]. Other modified versions of the assay include the combination with fluorescent in situ hybridization (FISH) [13,14,15] and the introduction of some adjustments to detect DNA crosslinks [16,17] and epigenetic modifications [18,19,20]. Furthermore, several high-throughput approaches have been recently described and developed [21,22,23,24,25].

The standard alkaline comet assay is widely used in basic research into DNA damage-repair mechanisms, for in vitro and in vivo genotoxicity testing, in ecotoxicology investigation, and in environmental, occupational, and dietary biomonitoring studies [9]. Although several cell types have been employed over years [26], including buccal [27,28,29], nasal [30] and lens epithelial cells [31,32] and sperm [33], the great majority of human studies have used—and still use—whole blood leukocytes [34,35,36,37,38,39] or isolated peripheral blood mononuclear cells—usually referred as peripheral blood lymphocytes (PBLs) [40,41,42], or both [43,44,45]. The use of PBLs as surrogate cells dominates the scene, as they circulate all over the body and have a long-life span, providing thereby information about the level of exposure and potential health risks [46]. Concurrently, the use of whole blood is also drawing attention, as the procedure requires smaller amounts of sample (a few microliters), shorter time, and seems to reduce baseline additional damage produced during the cell isolation process [44]. However, the standard procedure for obtaining blood samples could, in some cases, be perceived as invasive. To obtain PBLs, venous blood collection from the median cubital or antebrachial veins is usually carried out, while capillary blood samples are typically collected by lancet from the side of a finger. These sampling procedures could be cumbersome in children and/or poorly accepted by other subjects in the absence of risk perception. For these reasons, focusing on the collection of leukocytes from alternative specimens, as well as developing and optimizing non-invasive sampling procedures is a crucial issue. Buccal cells (BCs) are becoming increasingly popular in human biomonitoring studies, particularly because they can be obtained non-invasively, and have been used in several approaches to assess DNA damage by the comet assay [47,48]. However, BCs samples contain a mixed population of cells, mainly epithelial cells and leukocytes—but also erythrocytes and fibroblasts, with yields and proportions of cell types strongly depending on the isolation procedure and the physiological state of donors [49,50,51]. On this basis, isolation of buccal mononuclear leukocytes from saliva, whose collection is less invasive and better accepted by most of subjects, represents a potential strategy in human biomonitoring studies using the comet assay to assess DNA damage. Even if buccal mononuclear cells comprise lymphocytes (the largest majority, ~75%) and monocytes (~25%), as usually retrieved in the literature, hereinafter we will refer to buccal lymphocytes (BLs).

The use of BLs isolated by density gradient (DG) centrifugation in comet assay studies has been reported by several authors, laying the foundations for subsequent works. However, population characteristics (e.g., age, sex, etc.) have not always been investigated [51], or, in other cases, the number of non-exposed subjects was somehow limited [52,53]. Besides, some of those studies used cryopreserved samples [54,55] while others specifically focused attention in pre-school children [56].

In this context, the first aim of the present study was to set up and update a non-invasive procedure for the specific sampling and isolation of freshly collected BLs from saliva and to compare, in young subjects, baseline levels of DNA damage in BLs, leukocytes from fresh whole venous and capillary blood (WVB and WCB, respectively) with PBLs—the most frequently used surrogate cells for DNA damage assessment (hereinafter referred as Study 1).

Then, we used the developed procedure to assess baseline primary DNA damage in BLs in a wider population of young adults (hereinafter referred as Study 2). In addition, some population characteristics including sex, smoking habit, and body mass index (BMI) were also investigated and associated with DNA damage levels.

## 2. Materials and Methods

### 2.1. Study Population

A total of two different enrolments have been separately carried out. Firstly, the procedure set-up and comparisons between saliva and blood samples were carried out in a group of 15 young adults (Study 1) with ages ranging from 22 to 29 years. Subsequently, the assessment of primary DNA damage in BLs was carried out in a population of 54 individuals (Study 2) having ages ranging from 19 to 29 years. All the subjects enrolled were students at the University of Perugia (Italy) who participated in the study voluntarily. The participants were previously instructed on the nature of the study and gave their written informed consent. None of the participants evaluated wore orthodontic appliance or had been exposed to ionizing radiation and/or chemotherapy for at least 12 months before sampling. The ethical committee of the University of Perugia approved all experimental procedures of the study (protocol n. 2019-12R).

### 2.2. Questionnaire

In order to explore population characteristics, a specific questionnaire investigating sex, age, smoking habit, and anthropometric parameters was administered to all enrolled subjects.

### 2.3. Chemicals and Media

All reagents used were of analytical grade. Dulbecco’s phosphate-buffered saline (PBS), Minimum Essential Medium (MEM) and trypsin-EDTA were purchased from Corning (Glendale, AZ, United States of America). Lympholyte^®^ was obtained from Cerdelane (Burlington, NC, United States of America). Hydrochloric acid (HCl) was purchased from Thermo Fisher Scientific (Waltham, MA, United States of America). Acridine Orange (AO), 4′,6-diamidino-2-phenylindole (DAPI), ethidium bromide, low-melting point agarose (LMPA), normal-melting point agarose (NMPA), tris(hydroxymethyl)aminomethane (Tris) and Triton X-100 were obtained from Sigma-Aldrich Srl (Milan, Italy). Ethylenediaminetetracetic acid disodium (Na_2_EDTA) and tetrasodium (Na_4_EDTA) salt, sodium chloride (NaCl) and sodium hydroxide (NaOH) were purchased from Carlo Erba Reagenti (Milan, Italy). Dimethyl sulfoxide (DMSO) and ethanol absolute were purchased from AppliChem GmbH (Darmstadt, Germany).

### 2.4. Buccal Cells Collection and BLs Isolation

Saliva samples collection was always carried out in the morning (9–10 a.m.) between 8 May and 7 June 2019. Participants were previously asked not to eat prior to mouthwash collection. The method for collecting BLs followed previous procedures with modifications, mainly concerning centrifugation speed, times, and temperatures [51,52,53]. Participants were given two labelled sterile 50 mL conical polypropylene centrifuge tubes, each containing 20 mL cold (+4 °C) sterile saline solution (0.9% NaCl; pH 7.0). Volunteers stimulated and rinsed their mouths with saline solution twice for 1 min; mouthwashes were collected into another empty sterile 50 mL conical centrifuge tube. The samples were transported on ice to the laboratory and processed within 4 h of collection.

Mouthwashes were centrifuged for 8 min at 600× *g* (+4 °C). Supernatants were carefully removed, and cell pellets were re-suspended in 10 mL of PBS (pH 7.4). The samples were centrifuged again as above, the supernatants discarded, and cell pellets re-suspended in 6 mL of MEM (pH 7.4) with phenol red. BLs were isolated from the cell suspension by DG centrifugation using Lympholyte^®^ (pH 6.9), according to the manufacturer’s instructions. The presence of a non-colorless medium facilitates BLs identification in the next step, as they exactly locate at the interface between the medium and Lympholyte^®^ (colourless). Briefly, buccal cell suspension was carefully layered over 3 mL Lympholyte^®^ and centrifuged for 30 min at 800× *g* (+20 °C) (centrifuge brake turned-off). The upper layer was gently withdrawn, leaving the mononuclear cell layer undisturbed at the interface. BLs layer was removed and centrifuged at 1100× *g* for 10 min (+4 °C). After that, cell pellets were re-suspended in 1 mL PBS, and centrifuged again (1100× *g*, 8 min, +4 °C). Once supernatants were discarded, isolated BLs were re-suspended in PBS, and examined for number and viability through AO/DAPI assay. For each sample, 19 µL of cell suspension were mixed with 1 µL of AO/DAPI aqueous solution (AO: 30 µg/mL; DAPI: 100 µg/mL). A 10 µL aliquot of the resulting solution was loaded into the chambers NC-Slide A8™ and subsequently read for counting and viability using NucleoCounter^®^ NC-3000™ (ChemoMetec A/S, Allerød, Denmark). Then, the remaining cell suspensions were re-centrifuged (1100× *g*, 10 min, +4 °C), the supernatants removed, and finally cell pellets re-suspended in 0.7% LMPA (1 × 10^6^ cells in 300 µL LMPA) to continue with comet assay, basically following the original procedure [57] with minor modifications [58,59].

### 2.5. Blood Collection and PBLs Isolation

In addition to saliva samples collection, in order to compare baseline levels of primary DNA damage in leukocytes/lymphocytes obtained by adopting different sampling and processing procedures, 15 subjects were asked to undergo blood sampling. Whole blood contains between 5 and 10 × 10^6^ white blood cells (leukocytes)/mL and the largest fraction, ~60–70%, is represented by neutrophils (neutrophils, eosinophils, and basophils constitute the granulocytes/polymorphonuclear leukocytes); mononuclear cells (i.e., lymphocytes and monocytes) represent ~25–40% of white blood cells. The comet assay was then performed on PBLs following procedure (1), and on the complete spectrum of leukocytes following the procedures (2) and (3).

(1) PBLs—PBLs were collected and isolated from peripheral venous blood by DG centrifugation, a widely performed and standardized procedure Briefly, venous blood samples (4 mL) were firstly collected by venipuncture from the antecubital vein in Vacuette^®^ (Greiner Bio-One, Rome, Italy) vacuum tubes containing K_3_EDTA as anticoagulant. WVB aliquots were then diluted with an equal volume of PBS. An amount of 6 mL of diluted blood sample was layered over 3 mL of Lympholyte^®^ and centrifuged at 800× *g* for 30 min (+20 °C) (centrifuge brake turned-off). The upper layer was gently withdrawn, leaving the PBLs layer undisturbed at the interface. PBLs were removed, diluted with PBS and centrifuged at 300× *g* for 10 min (+20 °C). After that, cell pellets were re-suspended in 10 mL PBS and centrifuged again (200× *g*, 10 min, +20 °C). Once supernatants were discarded, isolated PBLs were re-suspended in 1 mL PBS and examined for number and viability. Finally, PBLs were re-suspended in 0.7% LMPA and layered onto pre-coated slides for the comet assay.

(2) Whole venous blood leukocytes (WVBLs)—a small aliquot (10 µL) of WVB sample collected as above was mixed with 100 µL LMPA and layered onto pre-treated conventional slides for direct investigation.

(3) Whole capillary blood leukocytes (WCBLs)—the same procedure was also applied to 10 µL of WCB obtained with a lancet by a puncture in the finger pad.

### 2.6. Comet Assay

The original three-layer procedure was conducted under alkaline conditions (alkaline unwinding/alkaline electrophoresis, pH > 13) [60]. To obtain microgels containing approximately 1 × 10^5^ cells, 30 µL of cell suspension in LMPA were pipetted onto 1% NMPA pre-coated conventional microscope slides. After microgels were allowed to spread using a cover slip, slides were kept on an ice-cold flat tray for 10 min for solidification. Then, cover slips were removed and 75 µL LMPA were added as a top layer. For microscope analysis, two slides per subject were prepared. Cells included in the agarose microgels were lysed at +4 °C in the dark overnight (10 mM Tris–HCl, 2.5 M NaCl, 100 mM Na_2_EDTA, 1% Triton X-100 added just before use; pH 10). After that, the DNA was allowed to unwind for 20 min in alkaline electrophoresis buffer (1 mM Na_4_EDTA, 300 mM NaOH, pH > 13) and subjected to electrophoresis in the same buffer for 20 min (1 V/cm and 300 mA). In blood samples processing procedure, 10% DMSO was also included in both lysis and electrophoresis solution to scavenge radicals generated by the iron released from hemoglobin. Lastly, the slides were neutralized with tris buffer (0.4 M Tris-HCl; pH 7.5) and the agarose microgels dehydrated by placing the slides in 70% ethanol for 5 min. All the steps for slide preparation were performed under yellow light to prevent additional DNA damage. To evaluate DNA damage, the slides were stained with ethidium bromide (50 µL, 20 µg/mL) and examined by using an Olympus BΧ41 (Japan) fluorescence microscope equipped with a high-sensitivity charge-coupled device (CCD) camera connected to a computerized image analysis system (“Comet Assay III”, Perspective Instruments Ltd., Suffolk, UK). A total of one hundred randomly selected cells per subject (two slides/subject, 50 comets/slide) were analysed [61]. Tail length (µm), tail intensity (%), and tail moment were chosen as damage parameters. The median of the scored comets for each slide was used to calculate the group means [62,63].

### 2.7. Statistical Analysis

Results are expressed as the mean ± standard deviation (SD) or standard error of the mean (SEM). The Kolmogorov–Smirnov test was used to verify the distribution of data, which were found to be parametric. One-way analysis of variance (ANOVA) with Dunnett’s post hoc test was used to compare primary DNA damage in PBLs, BLs, WVBLs, and WCBLs. Agreement between the damage parameters evaluated in PBLs (gold standard test) and DNA damage parameters evaluated in BLs, WVBLs and WCBLs was plotted using Bland-Altman method [64]. A linear multiple regression (LMR, block-wise) method was computed for DNA damage levels as dependent variables. The relative confounding factors (i.e., sex, smoking, and BMI) were examined in turn (Student *t*-test or ANOVA, Dunnett’s post hoc), before constructing a final LMR containing all potential confounders.

All statistical analyses were carried out with SPSS software for Windows (version 20.0; SPSS Inc., Chicago, IL, USA); *p*-values < 0.05 were considered as statistically significant.

## 3. Results

### 3.1. Population Characteristics

Population characteristics are summarized in Table 1. The Study 1 included 15 young volunteers (9 males and 6 females; age, group mean ± SD 24.4 ± 2.8). A smoking habit was present in 3 male subjects. Mean BMI (± SD) were 23.5 ± 2.6 for males, and 23.3 ± 4.9 for females.

The Study 2 included 54 young volunteers (14 males and 40 females; age, group mean ± SD 22.4 ± 1.7). A smoking habit was present in the 33.3% (*n* = 18) of the enrolled population. When considering BMI, 45 subjects out of 54 (83.3%) were normal weight; underweight (*n* = 2), and overweight/obese (*n* = 7) categories were also represented in the study population.

### 3.2. Study 1: Comparison of Primary DNA Damage Detected in PBLs, BLs, WVBLs and WCBLs

The first aim of this study (Study 1) was to update a method for BLs collection and isolation, and to compare primary DNA damage in BLs, WVBLs, and WCBLs with PBLs. We collected, on average, 4.9 × 10^5^ cells (BLs)/mL, characterized by a mean viability (%) of 77.8 ± 0.9 (Table S1). As these outcomes were well-suitable for successive passages, we further continued with comet assay performance in these cells. Figure S1 shows representative images from comet assay slides prepared following the above-described procedures. As shown in Table 2, among others, BLs showed the highest levels of basal primary damage, while PBLs showed the lowest values. Analysis of variance showed that tail length, tail intensity, and tail moment values in BLs are significantly higher compared with PBLs (*p* = 0.001). Moreover, tail intensity in WCBLs was higher than in PBLs by a factor of two (*p* = 0.008).

In the Bland-Altman method, the differences between values obtained from two techniques are plotted against the averages of the values of two techniques. Horizontal lines are fixed at the mean difference and at the limits of agreement, which are computed as the mean difference ±1.96 standard deviation. The value of 1.96 is set as 95% of the area of a normal distribution is included within 1.96 standard deviations of the mean. The Bland–Altman analysis (Figures S2–S4) indicates that the 95% limits of agreement between the tail intensity in BLs and the tail intensity in PBLs ranged from ₋5.66 to 0.19, while the mean of the difference between the two methods (bias) is −2.73, and the standard deviation of the bias (precision) is 1.49 (Table S2). This indicates that the two methods provide similar measures as the level of agreement lies within the accepted range.

### 3.3. Study 2: Primary DNA Damage Assessment in Isolated BLs

A further aim of this study was to assess baseline primary DNA damage in BLs in a wider enrolled population (*n* = 54). Mean values of tail length (µm), tail intensity (%) and tail moment in the recruited population are summarized in Table 3. Table 3 also shows the influence of sex, smoking and BMI on the comet assay descriptors in BLs. The level of DNA damage was similar in women and men, and in smokers and non-smokers. When the results were stratified by BMI, underweight subjects were not included in the statistical analysis because of the very small sub-group size.

Globally, linear multiple regression analysis (Table 4) showed that the extent of primary DNA damage in BLs is not influenced by sex, smoking habit, and BMI.

## 4. Discussion

As many subjects could perceive blood collection as cumbersome or too invasive, moving to alternative, better-accepted procedures for leukocytes collection might represent a crucial step in biomonitoring techniques improvement. In this context, this paper describes an updated procedure for the collection, isolation, and measurement of primary DNA damage in fresh BLs. First, we successfully yielded a suitable number of viable cells, widely sufficient to perform the comet assay. Overall, tail intensity detected in oral mononuclear leukocytes was comparable [56] or lower than that reported in other studies, in both control and exposed subjects [51,55]. Another work [52] used visual scoring approach, grading different levels of DNA damage from 0 to 4, where 4 was the most severe damage for each comet visualized. In that case, comet score ranged between 50 and 100 arbitrary units, which corresponded, according to a conversion curve obtained by regression analysis [65], to a range of estimated percentage of DNA in the tail of ca. 5.4 to 16.6. Likewise, primary DNA damage levels are partially superimposable or higher that those assessed in our study. En masse, these outcomes testify to a reduction in method-related aspecific damage and to an overall refinement of the procedure. Moreover, we used three different damage parameters—namely tail length, tail intensity and tail moment—in order to better elucidate any potential association and strengthen results interpretation.

In the first part of this study, we assessed and compared, in a group of 15 subjects, primary DNA damage in leukocytes obtained by different sampling and processing procedures, namely BLs, PBLs, WCBLs, and WVBLs. As expected, BLs showed lower viability and higher levels of primary DNA damage when compared with PBLs. Buccal leukocytes are directly exposed to air, beverages, and food passage through the mouth, representing appropriate target cells for the evaluation of their effects. However, direct exposure to potentially genotoxic compounds could result in higher levels of baseline damage. Moreover, higher aspecific damage might have been added during BLs sampling and processing procedures, in which a saliva sample is exposed to environmental stress for longer time compared with PBLs isolation procedure.

Even though results obtained using isolated PBLs and whole blood in different studies should not be directly comparable—whole blood contains the whole spectrum of leukocytes, not only lymphocytes—it could be useful to compare, in the same population, basal DNA damage among differently-collected blood specimens. Indeed, the choice of different blood-derived DNA source to be used in the comet assay could represent a non-negligible source of variability [61].

In our study, no statistically significant differences in terms of primary DNA damage were observed between freshly isolated PBLs and WVBLs. This is in line with other recent works [66,67]. Differently from our findings, another study revealed no differences between basal DNA damage between fresh whole blood collected by venipuncture and samples collected by lancet in the same donors [68]. Moreover, Sirota and Kuznetsova [69] found a significantly higher degree of DNA damage in venous blood samples compared with capillary blood samples. However, the authors justified this difference as a consequence of different temperature conditions of alkaline electrophoresis, rather than an actual sample-specific dissimilarity.

We finally hypothesized some potentially critical steps and issues that should be taken into serious consideration in the approach of this procedure:

- *Operator’s training*. The removal of the BLs layer after DG centrifugation is probably the most critical step of the whole procedure. Compared with the analogous step in PBLs isolation, BLs layer is relatively harder to identify and remove correctly and needs a preliminary training before performing it.
- *Number of samples processed per day*. If sampling procedure could be carried out in a single session, an excessive number of samples to be processed per day could introduce an aspecific and unmanageable bench-time induced variability. We suggest not to exceed 8–10 samples per day, and to process all the samples within 4 h of collection.
- *Laboratory’s room temperature*. In order to avoid sample degradation, affect cell viability and add aspecific damage, we suggest carrying out sampling and processing procedures when laboratory’s room temperature ranges between 18 and 25 °C.

As the level of agreement between BLs and PBLs lied within the accepted range, we enrolled a wider population (*n* = 54) to assess baseline DNA damage in BLs. To understand whether and to what extent some non-modifiable (i.e., sex) and modifiable (i.e., BMI and smoking habit) factors could affect baseline DNA damage in BLs, variables were associated with comet assay descriptors. Overall, our results showed that sex and tobacco smoking habits do not seem to affect basal level of primary DNA damage in isolated BLs in young healthy adults.

We did not find any significant difference in DNA damage in males and females. This is in line with most of available studies, in which no differences were reported between levels of DNA strand breaks, EndoIII- and Fpg-sensitive sites in white blood cells between women and men [43,70].

Tobacco use was also investigated in our study. Two works reported significantly higher tail intensity values in BLs in smokers than in non-smokers [51,55]. However, harmoniously with our work, McCauley and co-workers [54] reported that smoking habit was not significantly associated with higher tail length, tail intensity or tail moment in BLs. Actually, whilst tobacco smoke represents a complex mixture of genotoxic and carcinogenic agents, the association of primary DNA damage and smoking habit is still a matter of debate. Heterogeneity of findings are underlined and well described in two different reviews [43,71]. Some factors that may contribute to the discrepancies observed in different studies is passive smoking, which is often overlooked, and the misconsideration of electronic cigarette use, also able to cause DNA strand breaks in lymphocytes [72]. In our study, “smoking habit” did not include electronic cigarettes, as none of the participants used them.

By virtue of its association with higher DNA damage levels [73,74,75,76,77], wearing orthodontic appliance was chosen as one of the exclusion criteria for this study, while wearing a less invasive orthodontic retention wire was not. Unexpectedly, higher values of each damage parameter, as well as lower cell viability, were found in subjects wearing retention wire, compared with the rest of the recruited population (data not shown), even though the difference did not reach the statistical significance. In any case, we suggest this aspect should be taken into consideration among the exclusion criteria in future biomonitoring studies using buccal cells.

To the best of our knowledge, this is the first time that low BMI (mean BMI = 17.65) has been found to be associated with higher DNA damage measured with comet assay. Even though literature concerning slightly underweight subjects is still lacking, our results are in line with other findings reporting an overall oxidative stress imbalance in severely underweight subjects [78].

In an “obesogen” society, particularly in Western countries, health risks associated with underweight condition have been relatively overlooked over the past few decades. Although the dramatic influence of high BMI on human health is widely documented and accepted [79], several pieces of evidence show poor health outcomes, high mortality, and high risk of developing certain cancers in underweight subjects, as well [80,81,82,83,84,85,86]. In this context, our findings deserve deeper investigations in wider underweight populations, using, for instance, further genotoxicity assays.

The results of our work need to be interpreted within its limitations. First, convenience sampling among University of Perugia students was used in this study. Different groups of subjects (males vs. females; smokers vs. non-smokers; BMI groups) were neither numerically equi-represented, nor stratified according to general population structure. As a result, our findings might not be directly transferred to general population. Second, even though we managed to improve existing procedures in terms of basal method-induced DNA damage, statistical dispersion is still relatively high when considering tail intensity of BLs. This might be due to the characteristics of the sample itself, and to the relatively small size of the population enrolled in this study, as well.

## 5. Conclusions

In conclusion, we successfully managed to set up and update a totally non-invasive, rapid, and well-accepted procedure for the sampling and isolation of freshly collected BLs from saliva to be used in the alkaline comet assay. Our results showed that basal levels of primary DNA damage in BLs isolated from young adults are not affected by sex and tobacco smoking habit, making these cells a good model in biomonitoring studies investigating environmental or occupational exposure. The B-comet assay certainly needs to be further validated in wider populations; regardless, we actually believe that this procedure represents a useful and reliable tool for future studies in humans.

## Figures and Tables

**Table 1:** General characteristics of the study population.

Characteristics	Study 1		Study 2	
	Males	Females	Males	Females
*n* (%) ^1^	9 (60.0)	6 (40.0)	14 (25.9)	40 (74.1)
Age (years) ^2^	26.0 ± 3.0	22.0 ± 0.0	23.1 ± 2.3	22.2 ± 1.5
Smoking ^1^				
Non-smokers	6 (66.7)	6 (100.0)	10 (71.4)	26 (65.0)
Smokers	3 (33.3)	0 (0.0)	4 (28.6)	14 (35.0)
BMI ^2,3^	23.5 ± 2.6	23.3 ± 4.9	23.1 ± 2.9	21.9 ± 3.2
Underweight ^1^	0 (0.0)	0 (0.0)	0 (0.0)	2 (5.0)
Normal weight	8 (88.9)	3 (50.0)	11 (78.6)	34 (85.0)
Overweight/obese	1 (11.1)	3 (50.0)	3 (21.4)	4 (10.0)

^1^ Number of subjects and % (between brackets), respectively. ^2^ Group mean ± standard deviation. ^3^ Body mass index (BMI) was calculated as weight/height^2^ (kg/m^2^) and nutritional status was classified according to the following cut-points: underweight, BMI < 18.5; normal weight, 18.5 ≤ BMI ≤ 24.9; overweight/obesity, BMI ≥ 25.

**Table 2:** Basal level of primary DNA damage in human peripheral blood lymphocytes (PBLs), buccal lymphocytes (BLs), whole venous blood leukocytes (WVBLs) and whole capillary blood leukocytes (WCBLs) in 15 volunteers (Study 1).

DNA Damage Parameter ^1^	PBLs	BLs	WVBLs	WCBLs
Tail length (µm)	14.207 ± 0.094	20.565 ± 0.866 *	15.529 ± 0.194	15.649 ± 0.464
Tail intensity (%)	0.877 ± 0.086	3.611 ± 0.345 *	1.247 ± 0.087	1.765 ± 0.163 *
Tail moment	0.061 ± 0.007	0.475 ± 0.073 *	0.097 ± 0.009	0.147 ± 0.017

^1^ Group mean ± SEM. * ANOVA (Dunnett’s post hoc); *p* < 0.05. DNA damage in PBLs was considered as control.

**Table 3:** Extent of primary DNA damage in human buccal lymphocytes (BLs) based on sex, smoking habit, and body mass index (BMI).

Primary DNA Damage ^1^	Tail Length (µm)	Tail Intensity (%)	Tail Moment
Whole population (*n* = 54)	25.7 ± 0.9	6.7 ± 0.4	1.0 ± 0.1
Sex			
Males (*n* = 14)	24.1 ± 1.5	6.1 ± 0.7	0.9 ± 0.1
Females (*n* = 40)	26.2 ± 1.1	7.0 ± 0.4	1.1 ± 0.1
Smoking			
Non-smokers (*n* = 36)	25.2 ± 1.1	6.7 ± 0.5	1.0 ± 0.1
Smokers (*n* = 18)	26.5 ± 1.6	7.0 ± 0.5	1.1 ± 0.1
BMI			
Underweight (*n* = 2)	31.5 ± 2.2	11.6 ± 0.6	1.9 ± 0.04
Normal weight (*n* = 45)	25.5 ± 1.0	6.8 ± 0.4	1.0 ± 0.1
Overweight/obese (*n* = 7)	24.8 ± 2.9	5.4 ± 1.3	0.8 ± 0.3

^1^ Group mean ± standard error of the mean.

**Table 4:** Linear multiple regression (LMR) analysis between possible independent predictors and extent of primary DNA damage in human buccal lymphocytes (BLs).

Variable	B	95% CI	β	*p*
Tail length (µm)				
Sex	2.09	−2.02; 6.21	0.14	0.31
Smoking	1.54	−2.38; 5.45	0.11	0.43
BMI	0.57	−0.82; 1.96	0.12	0.42
Tail intensity (%)				
Sex	1.00	−0.79; −0.79	0.15	0.27
Smoking	0.42	−1.26; −1.26	0.07	0.62
BMI	0.23	−0.36; −0.36	0.11	0.43
Tail moment				
Sex	0.13	−0.28; 0.54	0.09	0.52
Smoking	0.15	−0.25; 0.56	0.11	0.46
BMI	0.05	−0.09; 0.19	0.11	0.46

## Supplementary Materials

The following are available online at https://www.mdpi.com/1660-4601/17/24/9234/s1, Figure S1: Microphotographs of cells after being processed for the comet assay: (a) buccal lymphocytes (BLs); (b) peripheral blood lymphocytes (PBLs); (c) whole venous blood leukocytes (WVBLs); and (d) whole capillary blood leukocytes (WCBLs). Cells were stained with ethidium bromide (50 µL, 20 µg/mL) and examined by using an Olympus BΧ41 (Japan) fluorescence microscope equipped with a high-sensitivity CCD (charge-coupled device) camera; Figure S2: Bland–Altman plots illustrating the agreement between the damage parameters evaluated in peripheral blood lymphocytes (PBLs; gold standard) and those evaluated in buccal lymphocytes (BLs), whole venous blood leukocytes (WVBLs), and whole capillary blood leukocytes (WCBLs); Figure S3: Bland-Altman plots illustrating the agreement between the damage parameters evaluated in peripheral blood lymphocytes (PBLs;gold standardtest) and those evaluated in whole venous blood leukocytes (WVBLs); Figure S4: Bland-Altman plots illustrating the agreement between the damage parameters evaluated in peripheral blood lymphocytes (PBLs;gold standardtest) and those evaluated in whole capillary blood leukocytes (WCBLs). Table S1: Yield, viability, and cell diameter of peripheralbloodlymphocytes(PBLs) and buccallymphocytes (BLs); Table S2: Bland-Altman analysis: DNA parameters (comet assay) evaluated in peripheral blood lymphocytes (PBLs); buccal lymphocytes(BLs); whole venous bloodleukocytes (WVBLs); whole capillary bloodleukocytes(WCBLs).
