# Supplementary material for: B-Comet Assay (Comet Assay on Buccal Cells) for the Evaluation of Primary DNA Damage in Human Biomonitoring Studies
Source: Int J Environ Res Public Health. 2020 Dec 10;17(24):9234. doi: 10.3390/ijerph17249234 (PMC7763633; doi:10.3390/ijerph17249234)
Supplement: Supplementary file 1 [file ijerph-17-09234-s001.pdf]

International Journal of
*Environmental Research and Public Health*

MDPI

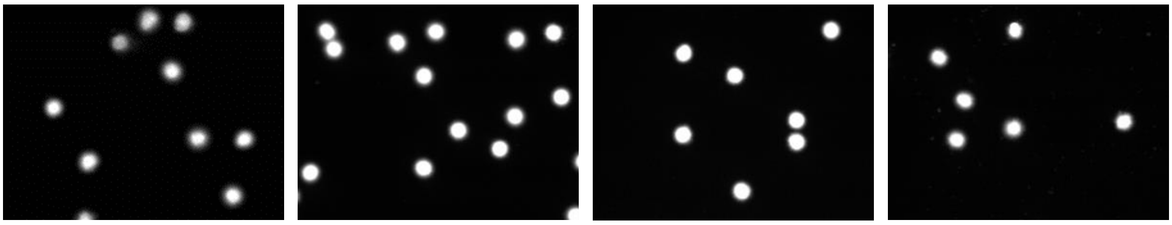

(**a**) (**c**) (**b**) (**d**)

**Figure S1.** Microphotographs of cells after being processed for the comet assay: (**a**) buccal lymphocytes (BLs); (**b**) peripheral blood lymphocytes (PBLs); (**c**) whole venous blood leukocytes (WVBLs); and (**d**) whole capillary blood leukocytes (WCBLs). Cells were stained with ethidium bromide (50 μL, 20 μg/mL) and examined by using an Olympus BX41 (Japan) fluorescence microscope equipped with a high-sensitivity CCD (charge-coupled device) camera.

International Journal of
*Environmental Research and Public Health*

MDPI

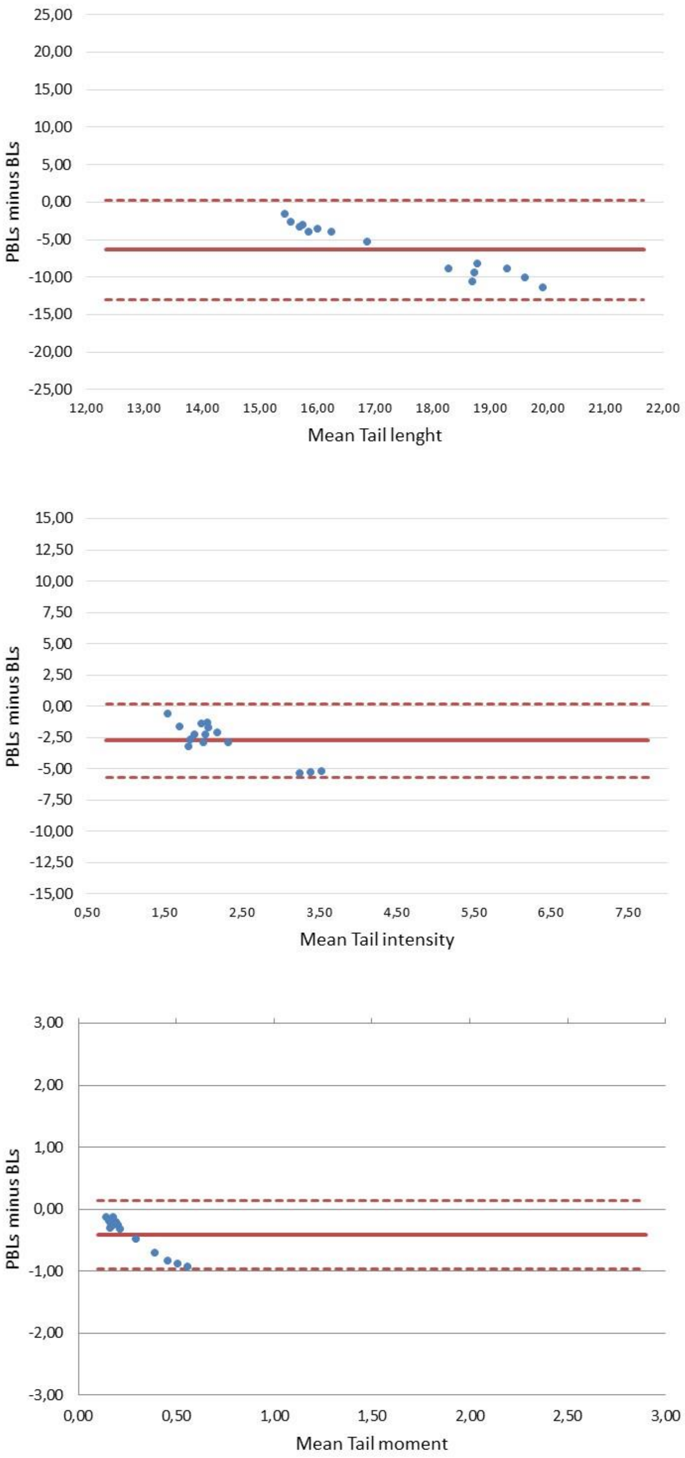


**Figure S2.** Bland-Altman plots illustrating the agreement between the damage parameters evaluated in peripheral blood lymphocytes (PBLs; gold standard test) and those evaluated in buccal lymphocytes (BLs).

International Journal of
*Environmental Research and Public Health*

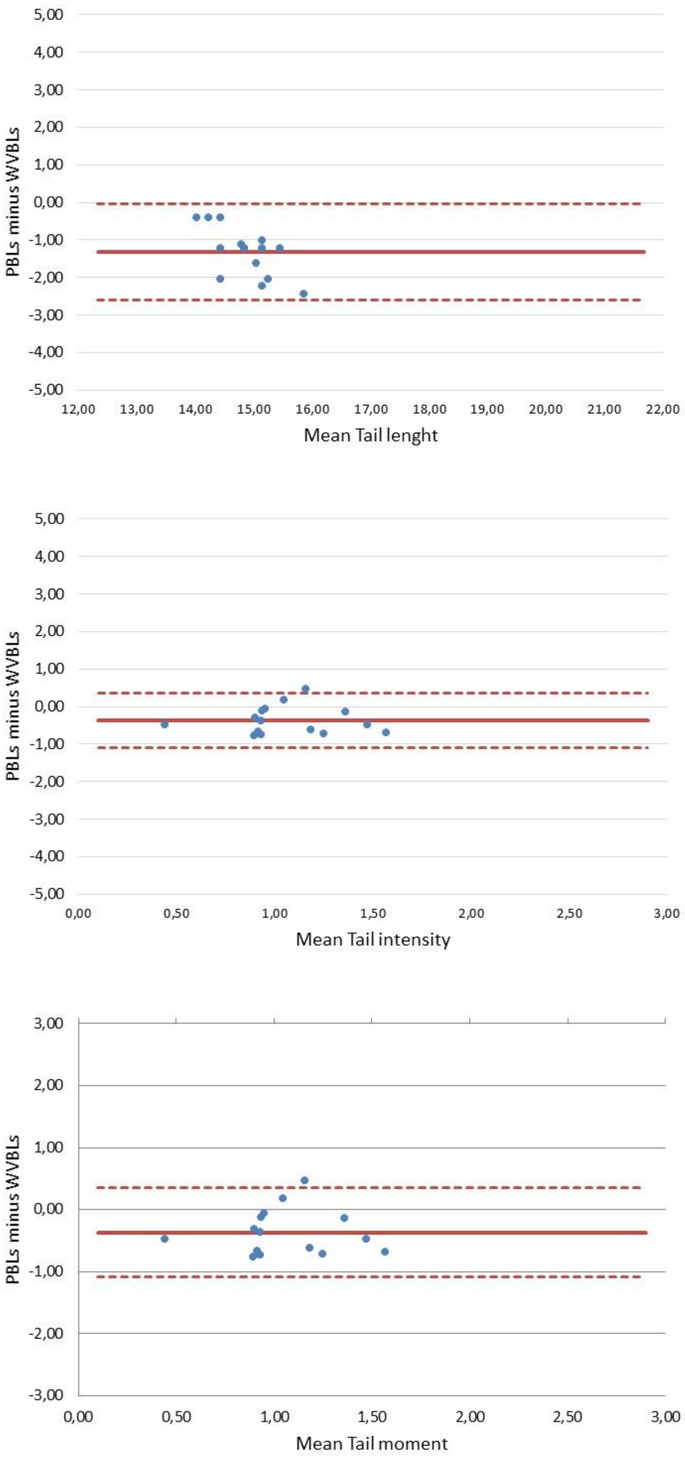


**Figure S3.** Bland-Altman plots illustrating the agreement between the damage parameters evaluated in peripheral blood lymphocytes (PBLs; gold standard test) and those evaluated in whole venous blood leukocytes (WVBLs).

International Journal of
*Environmental Research and Public Health*

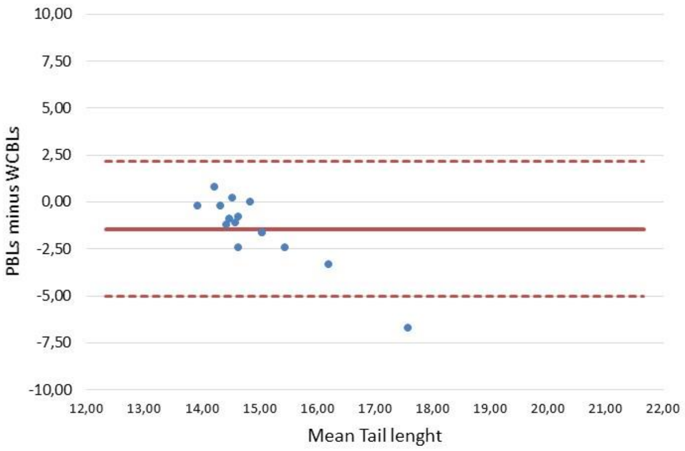


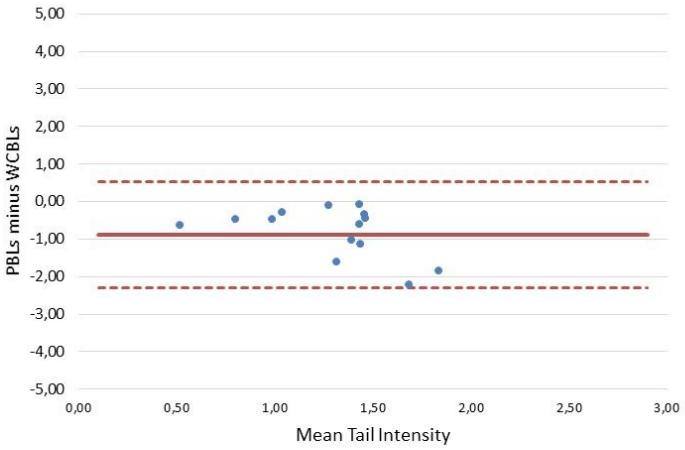


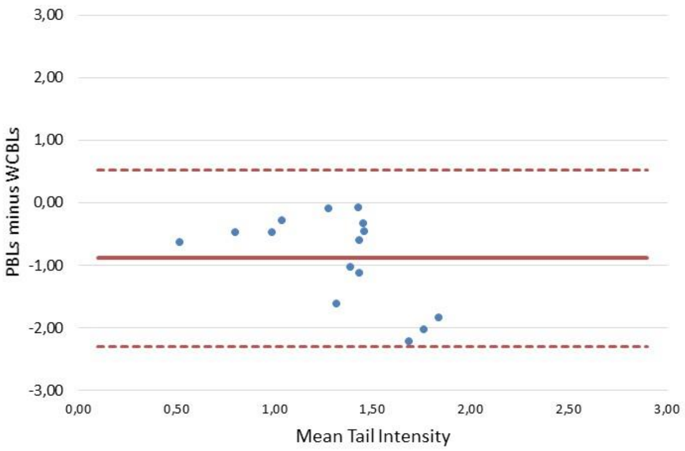


**Figure S4.** Bland-Altman plots illustrating the agreement between the damage parameters evaluated in peripheral blood lymphocytes (PBLs; gold standard test) and those evaluated in whole capillary blood leukocytes (WCBLs).

International Journal of
*Environmental Research and Public Health*

MDPI

**Table S1.** Yield, viability, and cell diameter of peripheral blood lymphocytes (PBLs) and buccal lymphocytes (BLs).

| | **PBLs** | **BLs** |
|---|---|---|
| Yield ($\times 10^5$/mL) [1] | 37.5 ± 5.9 | 4.9 ± 1.3 |
| Viability (%) [1] | 97.3 ± 0.3 | 77.8 ± 0.9 |
| Cell diameter (μm) [1] | 12.6 ± 0.4 | 11.33 ± 0.2 |

[1] Group mean ± standard error of the mean.

International Journal of
*Environmental Research and Public Health*

MDPI

**Table S2.** Bland-Altman analysis: DNA parameters (comet assay) evaluated in peripheral blood lymphocytes (PBLs); buccal lymphocytes (BLs); whole venous blood leukocytes (WVBLs); whole capillary blood leukocytes (WCBLs).

| | **PBLs *vs* BLs** | | | **PBLs *vs* WVBLs** | | | **PBLs *vs* WCBLs** | | |
|---|---|---|---|---|---|---|---|---|---|
| **Differences** | **TL** | **TI** | **TM** | **TL** | **TI** | **TM** | **TL** | **TI** | **TM** |
| Arithmetic mean (Bias) | -6.36 | -2.73 | -0.41 | -1.32 | -0.37 | -0.04 | -1.44 | -0.89 | -0.09 |
| 95% CI | [-8.23; -4.49] | [-2.73; -3.56] | [-0.57; -0.26] | [-1.68; -0.96] | [-0.57; -0.17] | [-0.06; -0.01 | [-2.46; -0.43] | [-1.28; -0.49] | -0.05 |
| Standard Error | 0.87 | 0.39 | 0.07 | 0.17 | 0.10 | 0.01 | 0.47 | 0.18 | 0.02 |
| Minimum | -11.38 | -5.36 | -0.94 | -2.44 | -0.77 | -0.09 | -6.07 | -2.21 | -0.20 |
| Maximum | -1.63 | -0.63 | -0.13 | -0.41 | 0.47 | 0.04 | 0.81 | -0.08 | -0.01 |
| Standard deviation (Precision) | 3.38 | 1.49 | 0.28 | 0.65 | 0.37 | 0.04 | 1.84 | 0.72 | 0.06 |
| Lower limit | -12.98 | -5.66 | -0.97 | -2.59 | -1.09 | -0.11 | -5.04 | -2.29 | -0.21 |
| Upper limit | 0.26 | 0.19 | 0.14 | -0.05 | 0.35 | 0.04 | 2.16 | 0.52 | 0.04 |

TL: tail length; TI: tail intensity; TM: tail moment

CI: confidence interval
